# High-Temperature Stealth Across Multi-Infrared and Microwave Bands with Efficient Radiative Thermal Management

**DOI:** 10.1007/s40820-025-01712-5

**Published:** 2025-03-24

**Authors:** Meng Zhao, Huanzheng Zhu, Bing Qin, Rongxuan Zhu, Jihao Zhang, Pintu Ghosh, Zuojia Wang, Min Qiu, Qiang Li

**Affiliations:** 1 State Key Laboratory of Extreme Photonics and Instrumentation, College of Optical Science and Engineering, Zhejiang University, Hangzhou, 310027 People’s Republic of China; 2 College of Information Science and Electronic Engineering, Zhejiang University, Hangzhou, 310027 People’s Republic of China; 3 Key Laboratory of 3D Micro/Nano Fabrication and Characterization of Zhejiang Province, School of Engineering, Westlake University, Hangzhou, 310024 People’s Republic of China

**Keywords:** Stealth, High temperature, Multispectral, Thermal management

## Abstract

High-temperature stealth is vital for enhancing the concealment, survivability, and longevity of critical assets. However, achieving stealth across multiple infrared bands—particularly in the short-wave infrared (SWIR) band—along with microwave stealth and efficient thermal management at high temperatures, remains a significant challenge. Here, we propose a strategy that integrates an IR-selective emitter (Mo/Si multilayer films) and a microwave metasurface (TiB_2_–Al_2_O_3_–TiB_2_) to enable multi-infrared band stealth, encompassing mid-wave infrared (MWIR), long-wave infrared (LWIR), and SWIR bands, and microwave (X-band) stealth at 700 °C, with simultaneous radiative cooling in non-atmospheric window (5–8 μm). At 700 °C, the device exhibits low emissivity of 0.38/0.44/0.60 in the MWIR/LWIR/SWIR bands, reflection loss below − 3 dB in the X-band (9.6–12 GHz), and high emissivity of 0.82 in 5–8 μm range—corresponding to a cooling power of 9.57 kW m^−2^. Moreover, under an input power of 17.3 kW m^−2^—equivalent to the aerodynamic heating at Mach 2.2—the device demonstrates a temperature reduction of 72.4 °C compared to a conventional low-emissivity molybdenum surface at high temperatures. This work provides comprehensive guidance on high-temperature stealth design, with far-reaching implications for multispectral information processing and thermal management in extreme high-temperature environments.

**Supplementary Information:**

The online version contains supplementary material available at 10.1007/s40820-025-01712-5.

## Highlights

- Simultaneous stealth across multiple infrared bands (short-wave infrared (SWIR), mid-wave infrared (MWIR), long-wave infrared (LWIR)) and microwaves at high temperatures (700 °C) is achieved.
- At 700 °C, the device features low emissivity of 0.38/0.44/0.60 in MWIR/LWIR/SWIR bands, reflection loss below − 3 dB in the X-band (9.6–12 GHz), and high emissivity of 0.82 in the 5–8 μm range.
- Under an input power equivalent to Mach 2.2 aerodynamic heating, effective thermal management achieves a 72.4 °C temperature reduction compared to conventional low-emissivity molybdenum.

**Supplementary Information:**

The online version contains supplementary material available at 10.1007/s40820-025-01712-5.

## Graphical abstract

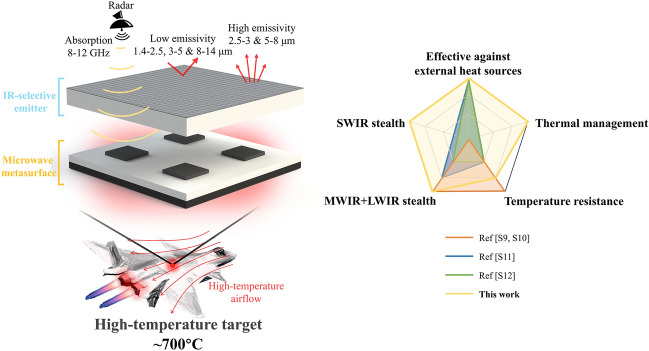

## Introduction

**Fig. 1:**
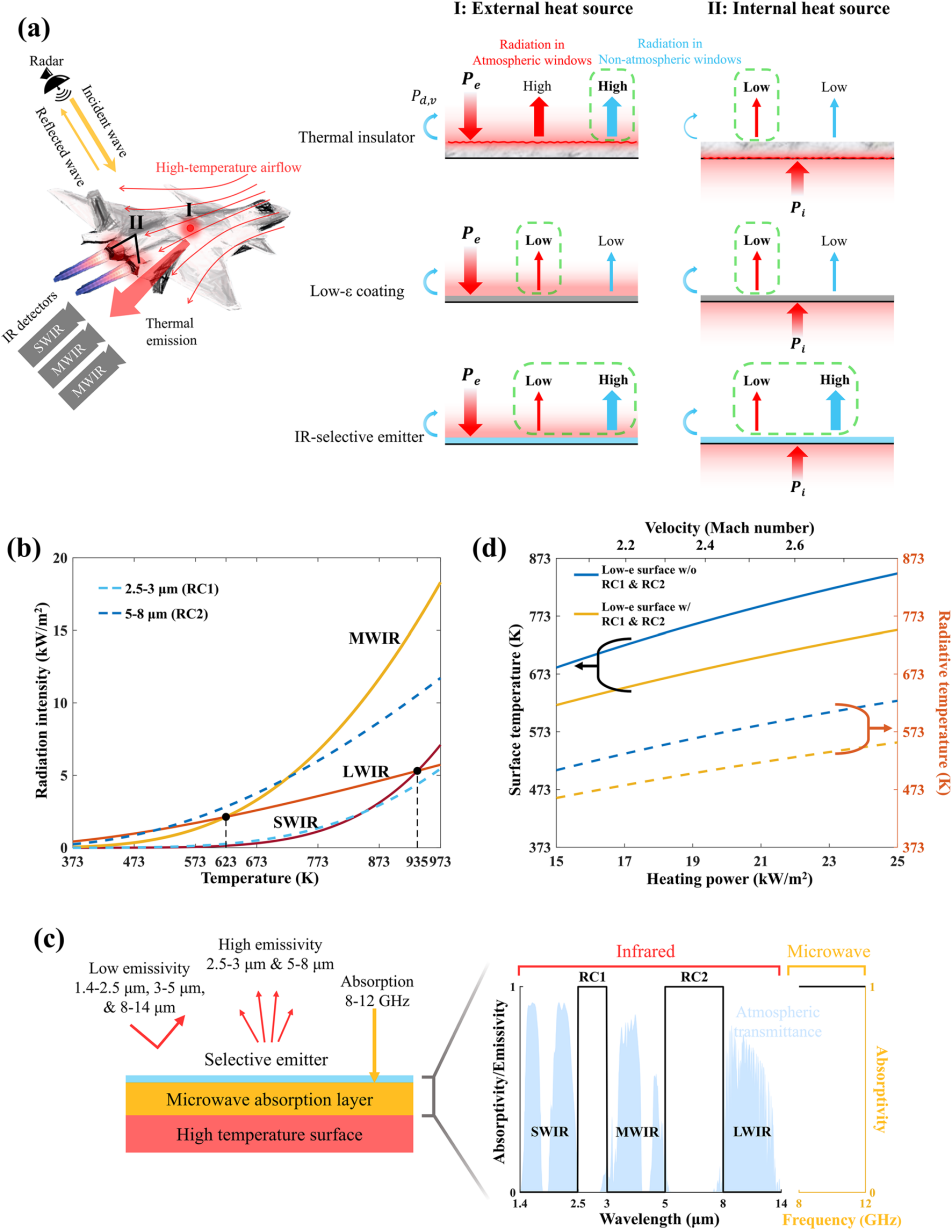
Schematic of high-temperature IR and microwave stealth with simultaneous thermal management. **a** Conceptual diagram of a high-temperature target detected by IR cameras (SWIR, MWIR, and LWIR) and radar systems (left). Regions I and II are heated by external and internal heat sources, respectively (with heating powers denoted as ${P}_{e}$ and ${P}_{i}$, and ${P}_{d, v}$ representing the heat conduction and convection power). A comparative analysis of various IR stealth strategies for regions I and II is shown on the right. The green dashed boxes represent superior performance. **b** Blackbody radiation intensity as a function of temperature in atmospheric windows (SWIR, MWIR, and LWIR) and radiative heat outlet bands (RC1 and RC2). **c** Ideal spectral characteristics for IR and microwave (X-band) stealth, shown alongside the atmospheric transmittance spectrum. **d** Surface temperature and radiative temperature variation with heating power and corresponding Mach number for the low-emissivity surface, with and without thermal management. (Color figure online)

Stealth technology [1–6] aims to conceal the characteristics of critical assets and render them invisible to various types of detectors, thereby enhancing survivability and longevity. On the modern battlefield, target detection primarily relies on the infrared (IR) and microwave bands (left panel of Fig. 1a). In the IR band, thermal cameras detect objects by capturing their emitted thermal radiation, with the radiation intensity proportional to the fourth power of the object’s temperature. In the microwave band, radar systems detect objects by emitting microwaves and receiving the reflected waves from the objects. With advancements in multispectral detection technology, multispectral stealth materials spanning visible to microwave spectra [2, 7–10] have flourished. Among these, IR-microwave compatible stealth materials are of critical importance [11–23]. Furthermore, as high-speed targets evolve, numerous critical assets (e.g., aircraft skin, converging nozzles) are affected by internal or external heat sources, resulting in high operating temperatures and intense thermal radiation. The existing multispectral stealth materials often fail at high temperatures [10–19], and their infrared stealth bands are typically limited to the conventional mid-wave infrared (MWIR, 3–5 μm) and long-wave infrared (LWIR, 8–14 μm) bands. However, at high temperatures, significant thermal radiation also occurs in the short-wave infrared (SWIR, 1.4–2.5 μm) band [24]. As the peak wavelength of thermal radiation blueshifts at elevated temperatures, objects become highly susceptible to detection. Therefore, simultaneous high-temperature IR and microwave stealth has become an imminent requirement.

Considerable efforts have been made toward achieving high-temperature stealth. High-temperature IR stealth can be achieved by reducing surface temperature through thermal insulators [25, 26]. This approach is effective against internal heat sources, but is accompanied by a heat accumulation issue. When exposed to external heat sources, it loses efficacy as the outer surface of thermal insulators can be directly heated to high temperatures (right panel of Fig. 1a). Another method for high-temperature IR stealth is emissivity modulation [27–31] through single-layer low-emissivity films (e.g., MXene [32], Au [33]) and Fabry–Pérot cavities [34]. However, these structures are incompatible with microwave stealth due to their high reflectivity to microwaves. High-temperature microwave stealth can be achieved through absorption induced by electric or magnetic losses [35–41], or scattering from destructive interference [42–44], but it conflicts with IR stealth because of high emissivity. High-temperature IR and microwave simultaneous stealth can be achieved by integrating thermal insulating layers with microwave absorbing components [13, 20, 21]; however, this method is prone to failure against external heat sources. An alternative approach involves combining microwave absorbers with frequency selective surfaces [21, 23], which feature low emissivity over a broadband IR spectrum (3–14 μm), but this suppresses radiative heat dissipation, leading to heat accumulation and thermal instability.

In conclusion, simultaneous IR and microwave stealth under high-temperature conditions faces several challenges: (1) Maintaining effective stealth under varying and complex heat sources, which requires considering the influence of heat source position on stealth performance. (2) Suppressing intense thermal radiation induced by high temperatures, with particular attention to the sharp increase in radiation intensity in the SWIR band. (3) Achieving effective thermal management under high temperatures, requiring the simultaneous achievement of both IR stealth and radiative heat dissipation. (4) Ensuring compatibility between IR and microwave stealth, demanding high reflection in the infrared band and low reflection in the microwave band.

In this work, the designed device, which combines an IR-selective emitter and a microwave metasurface, achieves stealth in multiple infrared bands (MWIR, LWIR, SWIR) and microwaves (X-band) at 700 °C, while also providing radiative cooling in the 5–8 μm range. At 700 °C, the device exhibits low-emissivity values of 0.38, 0.44, 0.60 in the MWIR, LWIR, and SWIR bands, respectively, reflection loss below − 3 dB in the X-band (9.6–12 GHz), and high emissivity of 0.82 in the 5–8 μm range—corresponding to a cooling power of 9.57 kW m^−2^. Furthermore, under an input power of 17.4 kW m^−2^, equivalent to the aerodynamic heating experienced by an aircraft flying at Mach 2.2, the device demonstrates a 72.4 °C reduction in temperature compared to a conventional low-emissivity molybdenum (Mo) surface at high temperatures.

## Experimental Section

### Simulation

The simulation of absorptance/emissivity spectra is conducted using the transfer matrix method and FDTD solutions. The electric field intensity distribution is calculated using FDTD solutions, and the refractive index and the extinction coefficient of the materials are provided in Fig. S6. Microwave simulations are performed using the radio frequency module (electromagnetic waves, frequency domain) in COMSOL Multiphysics. According to the measurement results in [37], the conductivity of TiB_2_ used in the simulations is 2.15 × 10^6^ S m^−1^, the relative permittivity of Al_2_O_3_ is 8.5 + 0.55i, and the relative permittivity of the high-temperature adhesive is 2 + 0.1i.

### Fabrication

All materials have a purity greater than 99.99%, ensuring the absence of impurities. The IR-selective emitter is fabricated by electron beam evaporation on Al_2_O_3_ substrate, with deposition rates of 1 Å s^−1^ for Ti, 1.5 Å s^−1^ for Mo, 3 Å s^−1^ for Si, and 1 Å s^−1^ for Al_2_O_3_ and a base vacuum of 1e-6 Torr. After deposition, the sample is annealed at 500 °C for 3 min in a nitrogen atmosphere. Finally, the surface is etched using a picosecond laser processing equipment (YLET3030UF). A 15 mm × 15 mm sample is also prepared for reflectivity and emissivity measurements. The TiB_2_ squares and the backplate are bonded to the Al_2_O_3_ intermediate layer using high-temperature adhesive (ARON CERAMIC-D/TOAGOSEI), with estimated thicknesses of 0.05 and 0.15 mm, respectively.

### Optical Characterization

The reflectivity spectrum in the spectral range of 1.4–14 μm is acquired by employing an Fourier transform infrared (FTIR) microscope (Hyperion 1000, Brucker) and an FTIR spectrometer (Vertex 70, Brucker), while utilizing an mercury cadmium telluride detector. For emissivity measurements, the FTIR spectrometer and FTIR microscope are employed, and the sample is heated from 200 to 800 °C on a high-temperature stage (Linkam TS1500), to probe the spectral range of 3–14 μm.

A Telops camera is used to measure the IR signal intensity in the MWIR band and the SWIR band (with a 1.5–2.5 μm band-pass filter). A Jenoptik camera is used to measure the radiative temperature in the LWIR band.

The average emissivity $\epsilon$ is given by:

1 $$\begin{array}{cc}& \\ {\int }_{\lambda 1}^{\lambda 2} u\left(\lambda ,{T}_{\text{r }}\right)\text{d}\lambda & ={\int }_{\lambda 1}^{\lambda 2} \epsilon u\left(\lambda ,{T}_{\text{s }}\right)\text{d}\lambda +{\int }_{\lambda 1}^{\lambda 2} r u\left(\lambda ,{T}_{\text{e }}\right)\text{d}\lambda \end{array}$$

where *u* is the blackbody radiation energy density, *T*_s​_ is the surface temperature, *T*_r​_ is the radiative temperature measured by the IR camera, *T*_e​_ is the ambient temperature, and *r* is the reflectivity. Since there is no transmittance, $r\approx 1-\epsilon$.

The measurements are taken in a laboratory under controlled conditions, with a constant temperature of 20 °C, a relative humidity of 40% RH, and standard atmospheric pressure.

### Surface Temperature and Microwave Measurement

Surface temperature measurement: The IR-selective emitter and Mo plate are placed on a heater powered by a current source, with insulating aerogel underneath to prevent heat conduction. The power density *P* is defined as the average power per unit area of the heating plate, calculated using the formula:

2 $$P=\frac{UI}{S}$$

where *U* and *I* represent the voltage and current output from the power source, respectively, and *S* denotes the area of the heating plate, which measures 120 cm × 120 cm. The heating wire primarily heats the upper surface of the thermal stage.

The surface temperature is measured by a thermocouple (KPS-IN600-K) secured with a C-clamp. Each temperature data point was obtained after a prolonged waiting period to ensure the thermocouple readings stabilized, with fluctuations confined to within ± 0.1 °C. The infrared camera was positioned 80 cm above the sample and automatically focused on the sample surface. Due to the relatively short measurement distance, the recorded radiation temperatures are accurate.

The microwave absorption performance is characterized using a Vector Network Analyzer (N5227B) and an NRL-arc system (Fig. S3c) in a microwave anechoic chamber. The microwave reflectivity *R* at normal incidence can be measured, and the reflection loss RL is calculated as:

3 $$\text{RL}=10\text{log}R$$

The bandwidth of the frequency range where the $\text{RL}<-3\text{ dB}$ is defined as the effective absorption bandwidth.

## Results and Discussion

### Principle for High-Temperature Multi-Infrared and Microwave Stealth

A high-temperature object may be subjected to heating from external heat sources such as aerodynamic heating in region I (left panel of Fig. 1a), characterized by a power input ${P}_{\text{e}}$, or from internal heat sources, such as exhaust plume heating in region II (left panel of Fig. 1a), characterized by a power input ${P}_{i}$. Simultaneously, the object dissipates heat through convection and conduction, represented by power output ${P}_{d, v}$ (right panel of Fig. 1a). Thermal insulators tend to fail in IR stealth against external heat sources, as their outer surfaces can be directly heated to high temperatures. Although low-emissivity coatings can effectively suppress thermal radiation over a broadband IR spectrum, they can result in thermal instability. In contrast, IR-selective emitter enables substantial heat dissipation through radiative cooling in non-atmospheric windows while maintaining IR stealth even at high temperatures.

To assess the relative importance of the three atmospheric windows (SWIR, MWIR, and LWIR) for stealth and the radiative cooling capability in non-atmospheric windows at elevated temperatures, the radiation intensity of a blackbody is analyzed across a temperature range of 373–973 K. Figure 1b illustrates the radiation intensity in SWIR, MWIR, and LWIR bands, as well as two radiative cooling bands (2.5–3 and 5–8 μm, referred to as RC1 and RC2). When the temperature exceeds 623 K, the radiation intensity in the MWIR band surpasses that of the LWIR band. As the temperature continues to rise to 935 K, the radiation intensity in the SWIR band also exceeds that of the LWIR band, emphasizing the importance of suppressing shorter-wavelength radiation for effective high-temperature IR stealth. For a blackbody at 973 K, 36.1% of the power is radiated in the MWIR band, while 14.0% and 11.3% in the SWIR and LWIR bands, respectively. This indicates that, for high-temperature objects (~ 700 °C), the MWIR band is of critical importance for stealth, and the SWIR band exhibits considerable radiation intensity that requires suppression. Additionally, the radiation intensity in the two radiative cooling bands (RC1 and RC2) is quite significant. For instance, at 973 K, an object can achieve a theoretical limit for radiative heat dissipation of approximately 17.2 kW m^−2^ by combining the power contributions from RC1 and RC2. This indicates that thermal management through radiative heat dissipation in the non-atmospheric windows holds immense potential for cooling high-temperature objects.

Based on the above analysis, the ideal spectrum for high-temperature IR and microwave stealth is shown in Fig. 1c. The IR-selective emitter exhibits low emissivity in the MWIR, LWIR, and SWIR atmospheric windows, but high emissivity in RC1 and RC2. Meanwhile, microwaves (X-band) pass through the IR-selective emitter and are absorbed by the underlying layer.

To see how thermal management reduces surface temperature and improves IR stealth, we calculated the surface and radiative temperatures of a low-emissivity surface (*ε* = 0.3) with and without RC1 and RC2 under different heating powers (Fig. 1d). The surface temperature is calculated using the following thermal equilibrium equation:

4 $${P}_{\text{e}/\text{i}}={P}_{\text{d},\text{ v}}\left(h,{T}_{s}\right)+{P}_{r}\left(\varepsilon (\lambda ),{T}_{s}\right)-{P}_{r,\text{ a}}$$

where ${P}_{\text{e}/\text{i}}$ represents the heating power from internal or external heat sources. The term ${P}_{\text{d},\text{ v}}$ is the heat dissipation power through conduction and convection, which correlates positively with the equivalent heat transfer coefficient $h$ and surface temperature ${T}_{s}$. Thermal radiation power, ${P}_{r}\left(\varepsilon (\lambda ),{T}_{s}\right)$, was calculated by integrating energy density provided by Plank’s law over all wavelengths, which depends on the emissivity distribution $\varepsilon (\lambda )$ and ${T}_{s}$. The term ${P}_{r, a}$ refers to the absorbed environmental radiation, which has minimal impact at elevated temperatures. The relationship between heating power and corresponding flight velocity (Mach number) is also calculated (Fig. 1d) based on the surface temperature–Mach number correlation provided in [45] (at an altitude of 100,000 feet and surface emissivity of 0.5) and the surface thermal equilibrium equation, Eq. (4). At a heating power of 15 kW m^−2^, the temperature of the low-emissivity surface with thermal management is 619 K, 65 K lower than that of the surface without thermal management. This temperature difference widens with increasing heating power; at 25 kW m^−2^, the temperature of the former reaches 749 K, 98 K lower than the latter. Consequently, the radiative temperature of the surface with thermal management is also noticeably lower. At 25 kW m^−2^, its radiative temperature is 555 K, 72 K lower than the latter. These results demonstrate that thermal management can substantially improve IR stealth performance by lowering surface temperature.

### Structure Design and IR Measurement

**Fig. 2:**
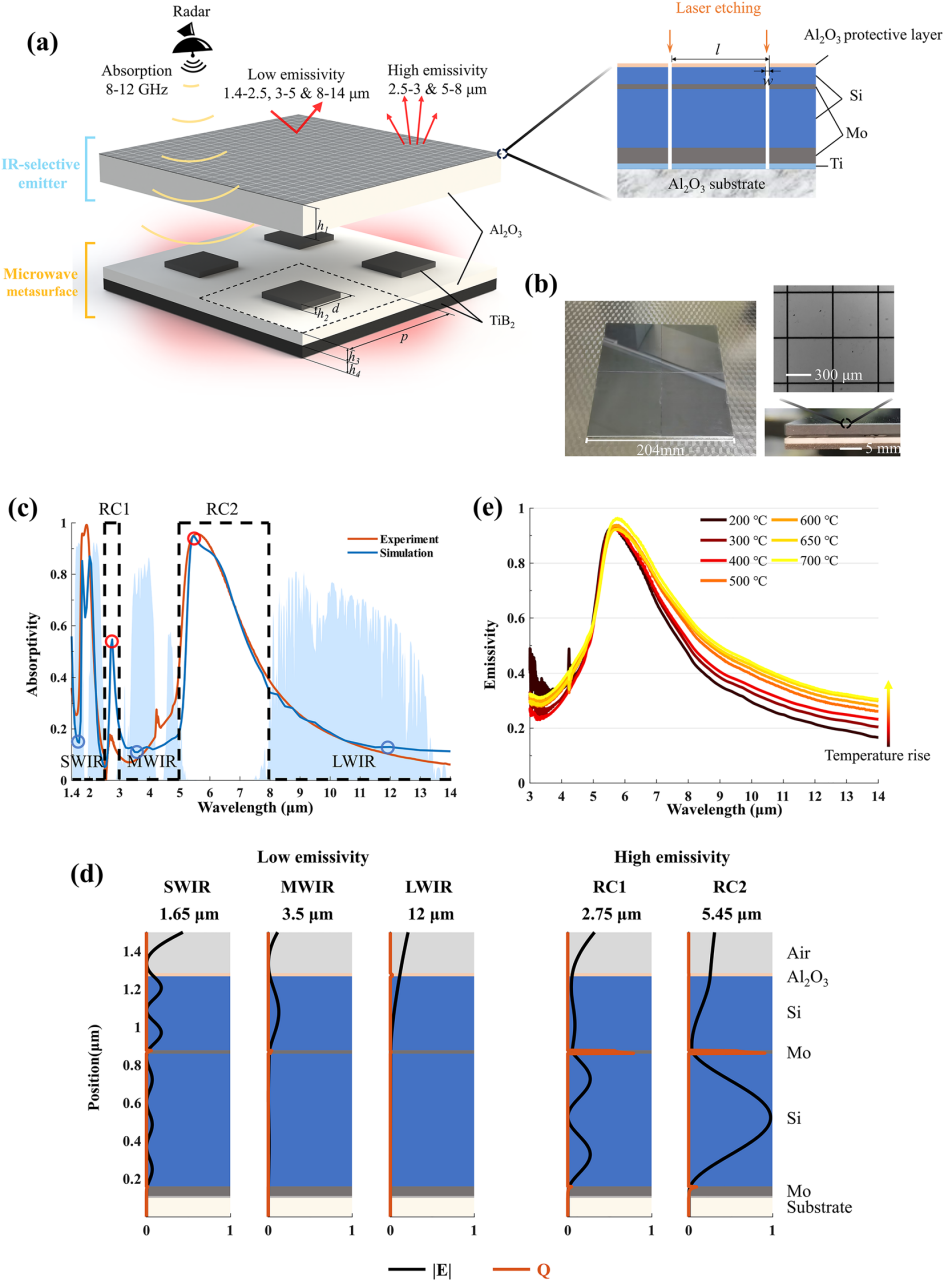
Structure, simulation, and characterization of the IR-selective emitter. **a** Schematic of the high-temperature IR and microwave stealth device, comprising an IR-selective emitter and a microwave metasurface. **b** Photographs of the fabricated sample, including top and side views, along with a microscope image of the laser-etched IR-selective emitter. **c** Measured and simulated absorptivity spectra of the IR-selective emitter. **d** Simulated electric field intensity (|*E*|, black lines) and resistive loss (*Q*, orange lines) distribution at representative wavelengths in low-emissivity bands (blue circles in **c**) and high-emissivity bands (red circles in **c**). **e** Measured emissivity spectra of the IR-selective emitter across a temperature ranging from 200 to 700 °C. (Color figure online)

The designed structure for high-temperature multi-infrared and microwave stealth is composed of an IR-selective emitter integrated with a microwave metasurface, as illustrated in Fig. 2a. The IR-selective emitter, consisting of a multilayer film of Al_2_O_3_ (17 nm)/Si (390 nm)/Mo (18 nm)/Si (700 nm)/Mo (50 nm)/Ti (10 nm), is deposited on four Al_2_O_3_ substrates, each measuring 102 mm × 102 mm and having a thickness of *h*_1_ = 2.3 mm. The top layer of Al_2_O_3_ serves as a protective layer with a dense structure, aiming to prevent moisture from penetrating the multilayer film and causing defects when moisture evaporated at high temperatures. The bottom layer of Ti functions as an adhesion layer, enhancing the bonding strength between the multilayer film and the substrate. The number of film layers is optimized because the thermal stress increases significantly with temperature, which can lead to failure at elevated temperatures. The multilayer film is then laser-etched with line divisions, with a period of *l* = 500 μm and a line width of *w* = 15 μm (microscope image in Fig. 2b). The laser etching process exhibits high accuracy, with negligible impact on structural properties such as surface roughness. It allows for microwave transmission without compromising infrared performance (Supplementary S1 and Fig. S1). After parameter optimization (Supplementary S2 and Fig. S2), the microwave metasurface beneath the IR-selective emitter consists of a periodic array of TiB_2_ square blocks (period *p* = 12 mm, side length *d* = 4.5 mm, and thickness *h*_2_ = 0.5 mm), an Al_2_O_3_ intermediate layer with thickness *h*_3_ = 1 mm, and TiB_2_ reflective backplate with thickness *h*_4_ = 1 mm. The metasurface achieves absorption through impedance matching in the X-band, and the reflection loss is measured (Fig. S3a). The components are bonded using high-temperature adhesive (Fig. S3b). The top and side views of the fabricated overall structure are shown in Fig. 2b. All the materials used in this structure (Si, Mo, Ti, TiB_2_, and Al_2_O_3_) exhibit excellent thermal stability at high temperatures.

The simulated and measured absorptivity spectra of the IR-selective emitter are shown in Fig. 2c. The electric field intensity |*E*| and resistive loss *Q* distributions at representative wavelengths corresponding to low emissivity (1.65, 3.5, and 12 μm, blue circles) and high emissivity (2.75 and 5.45 μm, red circles) are also calculated (Fig. 2d). The bottom Mo layer (50 nm) serves as a metallic reflective backplane, ensuring minimal infrared transmission. The intermediate Si layer (700 nm) is designed with an optical thickness near the 1st- and 2nd-order Fabry–Pérot resonance wavelengths (one half-wavelength at 5.45 μm and two half-wavelengths at 2.75 μm, Fig. 2d). This enables multiple reflections of light at the metal interfaces, thereby enhancing absorption in the RC1 and RC2 bands. The intermediate Mo layer (18 nm) is designed to partially transmit light in the RC1 and RC2 bands to enable resonant absorption, while reflecting light in the atmospheric window bands to achieve low emissivity. The upper Si layer (390 nm) with an optical thickness near a quarter-wavelength of 5.45 μm, acts as an anti-reflection coating to further enhance absorption in RC2 band. In SWIR, MWIR, and LWIR bands (1.65, 3.5, and 12 μm), light cannot interfere constructively within the multilayer film and is reflected back almost without loss. The average emissivity values of the selective emitter at room temperature in the SWIR, MWIR, and LWIR bands are 0.20, 0.24, and 0.16, respectively, which indicate superior IR stealth capability. In the two radiative cooling bands, 2.5–3 and 5–8 μm, the selective emitter shows the average emissivity values of 0.12 and 0.67, respectively. Despite the lower emissivity peak in 2.5–3 μm, the selective emitter still possesses excellent heat dissipation capability due to the high emissivity in the main radiative cooling band 5–8 μm.

To test the high-temperature performance, the emissivity of the IR-selective emitter is measured from 200 to 700 °C in the 3–14 μm range (Fig. 2e). As the temperature increases, the emissivity in the 3–14 μm range tends to rise steadily. At 700 °C, the average emissivity values of the selective emitter in the MWIR and LWIR bands are 0.38 and 0.44, respectively. Additionally, the selective emitter achieves an average emissivity of 0.82 in the 5–8 μm band, corresponding to a significant heat dissipation power of 9.57 kW m^−2^. The emissivity spectrum at temperatures above 700 °C is also measured (Fig. S4), and the average emissivity for MWIR, LWIR, and the 5–8 μm is calculated at various measurement temperatures (Table S1), which indicates that the IR-selective emitter degrades at 750 °C (Fig. S4). This is primarily attributed to the following factors: the change in the crystal structure of the multilayer film at high temperatures, the enhanced diffusion of oxygen leading to the oxidation of Mo, and the accumulation of thermal stress causing structural damage.

### High-Temperature MWIR/LWIR/SWIR Stealth

**Fig. 3:**
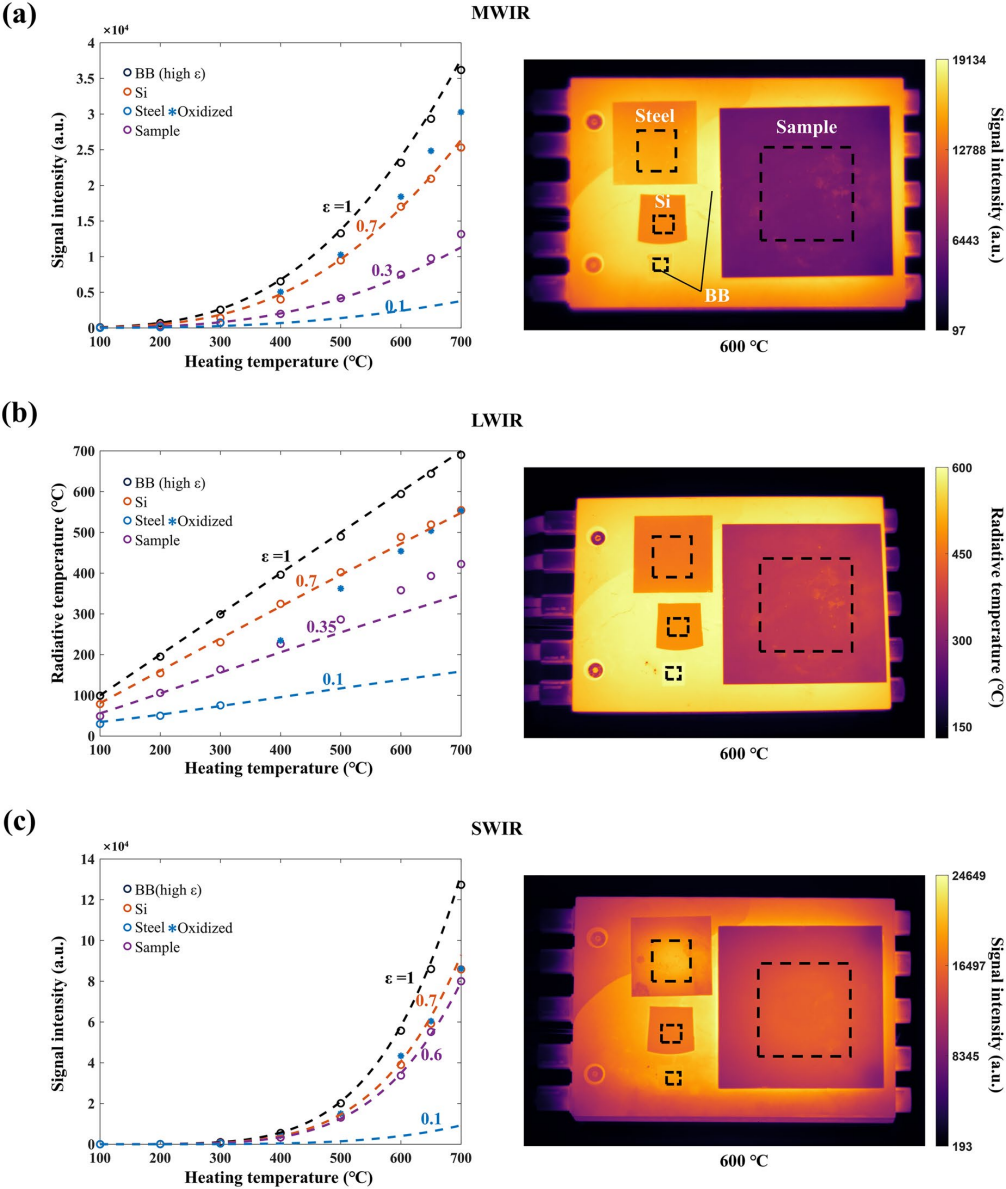
Demonstration of high-temperature stealth across multiple infrared bands (SWIR, MWIR, and LWIR). The IR-selective emitter is placed on a high-temperature ceramic heating stage, treated as a blackbody (BB), alongside a steel plate, a silicon wafer, and a BB reference, and heated to 700 °C. **a** Left panel illustrates the radiative signal intensity variation of the IR-selective emitter, steel, silicon wafer, and BB across different heating temperatures in the MWIR band, while the right panel presents a thermal image captured at 600 °C. **b** Left panel shows the radiative temperature variation of the IR-selective emitter, steel, silicon wafer, and BB in the LWIR band, with the right panel displaying a thermal image at 600 °C. **c** Left panel depicts the radiative signal intensity variation of the IR-selective emitter, steel, silicon wafer, and BB across different heating temperatures in the SWIR band, with the right panel showing a thermal image at 600 °C. Circles of different colors represent the measured average radiative signal intensity or radiative temperature of the IR-selective emitter, steel, silicon wafer, and BB at 100, 200, 300, 400, 500, 600, 650, and 700 °C. The blue asterisks indicate significant oxidization of the steel. The dashed lines represent the numerically calculated results of the radiative signal intensity or radiative temperature for various average emissivity values (0.1, 0.3, 0.35, 0.6, 0.7, and 1). (Color figure online)

High-temperature IR stealth is demonstrated using thermal imagers to observe the IR-selective emitter placed on a high-temperature heating stage, with a steel plate, a silicon wafers (Si), and a blackbody (BB) serving as references. Steel represents conventional low-emissivity materials, while silicon stands for conventional high-temperature resistant materials. The surface of the heating stage can be considered a blackbody. Heating temperatures are varied from 100 to 700 °C to assess IR stealth performance in MWIR, LWIR, and SWIR bands. Thermal images in the MWIR, LWIR, and SWIR bands on a heating stage at 600 °C are shown in the right panel of Fig. 3 (see Fig. S5 for other temperatures). By capturing and averaging the IR signals within the black dashed area, the radiation signal intensity in the MWIR band (left panels of Fig. 3a), the radiative temperature in the LWIR band (left panel of Fig. 3b), and the radiation signal intensity in the SWIR band (left panels of Fig. 3c) of the IR-selective emitter, steel plate, Si, and BB are plotted over a temperature range of 100–700 °C. The dashed lines represent the theoretically expected signal intensity or radiative temperature, calculated numerically for objects with certain emissivity *ε*. These curves corresponding to *ε* = 0.1, 0.3, 0.35, 0.6, 0.7, and 1 are used to compare experimental data with calculated results to estimate the emissivity values of the selective emitter and references (steel, Si, and BB).

In the MWIR band (Fig. 3a), the signal intensity of the IR-selective emitter is slightly higher than that of steel from 100 to 300 °C. However, when the temperature exceeds 300 °C, steel undergoes significant oxidation (blue asterisks), resulting in a sharp increase in its thermal emission. The IR-selective emitter (*ε* ~ 0.3) exhibits the lowest signal intensity, significantly below that of the BB (*ε* ~ 0.95), oxidized steel (*ε* > 0.7), and Si (*ε* ~ 0.7). Even at 700 °C, the signal intensity of the selective emitter remains 63.6% lower than that of the BB radiation, 56.5% lower than that of the steel plate, and 48.0% lower than that of the silicon wafer. The minimal signal intensity of the IR-selective emitter, when compared to the conventional low-emissivity materials, underscores its superior high-temperature stealth performance in the MWIR band.

In the LWIR band (Fig. 3b), the radiative temperature of the IR-selective emitter at 300 °C (*ε* ~ 0.35) is 163.6 °C, which is 135.4 °C lower than that of the BB and 66.7 °C lower than that of the Si wafer. Only the unoxidized steel shows a lower radiative temperature than the selective emitter, which is 75.5 °C. As temperature increases, the emissivity of the selective emitter in LWIR band slightly rises. This is mainly due to the enhanced lattice vibrations at high temperatures and the blue shift of the emission peak, which increases the proportion of shorter-wavelength radiation. However, even at 700 °C, the radiative temperature of selective emitter is 422.3 °C, which is 268.0 °C lower than that of the BB, 132.2 °C lower than that of the Si, and 131.1 °C lower than that of the steel plate. The lower radiative temperature of the IR-selective emitter compared to the conventional low-emissivity materials indicates its excellent stealth capability in the LWIR band.

In the SWIR band (Fig. 3c), the signal intensity from 100 and 300 °C is significantly lower compared to high temperatures. This phenomenon is consistent with the theoretical radiation intensity curve (Fig. 1b), as this band is farther from the peak radiation wavelength at lower temperatures. The signal intensity of the IR-selective emitter is higher than that of the steel plate but lower than that of the BB and Si wafer (Fig. S5). As the temperature increases, the signal intensity rises sharply. The IR-selective emitter (*ε* ~ 0.6) shows the lowest signal intensity, lower than blackbody (*ε* ~ 0.95), oxidized steel (*ε* ~ 0.7), and silicon (*ε* ~ 0.7) over a temperature range of 400 to 700 °C. At 700 °C, the signal intensity of the IR-selective emitter remains 37.2% lower than that of the BB, 7.1% lower than that of the steel plate, and 6.8% lower than that of the Si wafer. The lower radiative signal intensity of the IR-selective emitter compared to the low-emissivity references—especially at elevated temperatures—highlights its potential for stealth applications in the SWIR band.

### Demonstration of Thermal Management

**Fig. 4:**
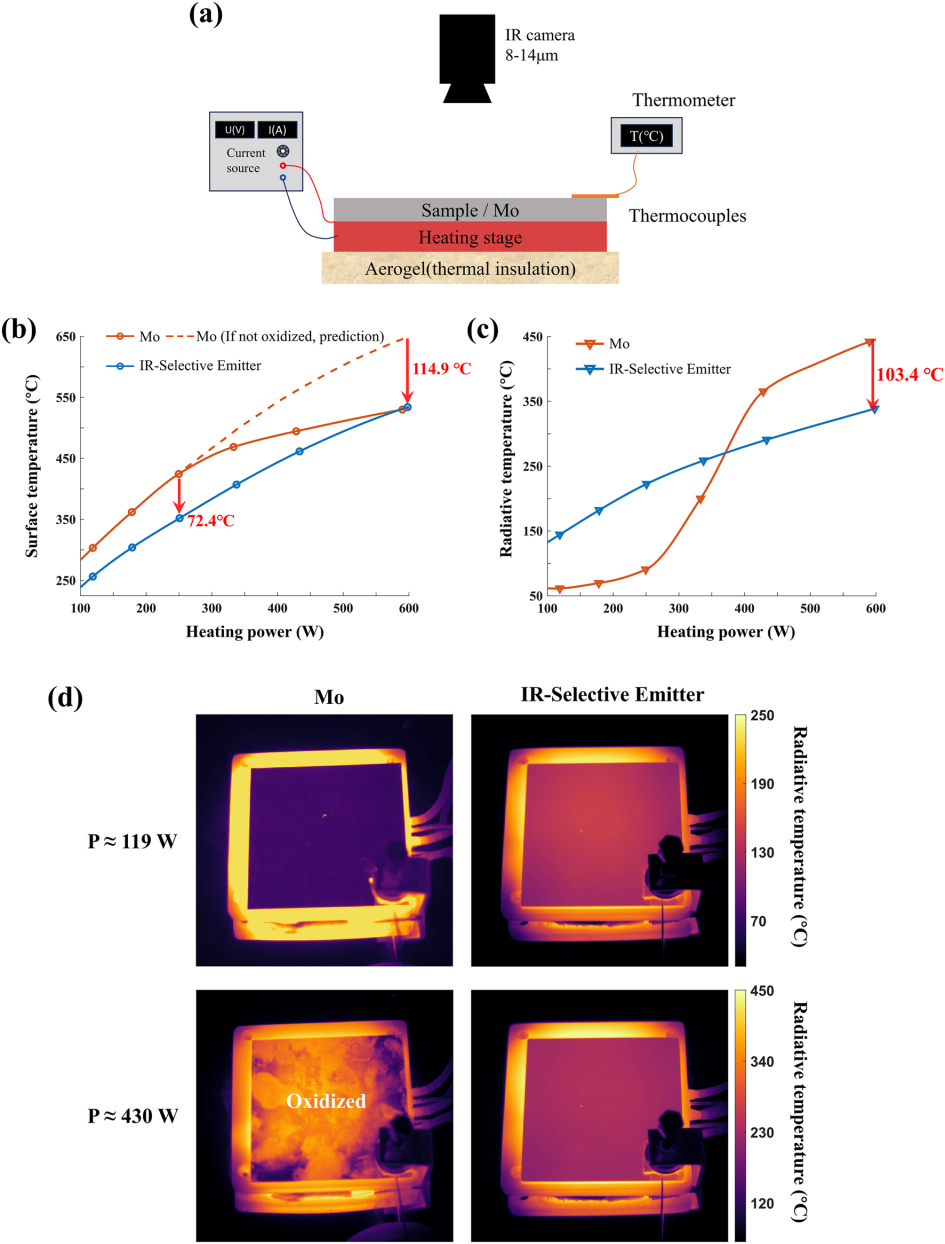
Surface temperature reduction and enhanced IR stealth performance enabled by thermal management. **a** Schematic of the experimental setup used to measure surface temperature and radiative temperature. **b** Measured surface temperature of the IR-selective emitter and Mo plate as the input power increases from 119 to 598 W. **c** Radiative temperature variation of the IR-selective emitter and Mo plate in the LWIR band. The dashed line represents the predicted surface temperature of Mo if it maintains a low emissivity. **d** Thermal images of the IR-selective emitter and Mo plate at the input power values of 119 and 430 W

To investigate the surface temperature reduction and IR stealth performance improvement, the surface and radiative temperatures of the IR-selective emitter and a molybdenum (Mo) reference plate of the same size are measured at thermal equilibrium under constant input powers, using the setup shown in Fig. 4a. Here, metal Mo is selected as the reference due to its low emissivity and excellent thermal stability, making it a widely used high-temperature IR stealth material [46]. At the same input power, the IR-selective emitter achieves a significant temperature reduction compared to the low-emissivity Mo plate (Fig. 4b). At an input power of 250 W (equivalent to a power density of 17.3 kW m^−2^), the IR-selective emitter exhibits a temperature of 352 °C, which is 72.4 °C lower than that of the Mo plate. The estimated power corresponds to the aerodynamic heating experienced by an aircraft flying at Mach 2.2 (Fig. 1d), indicating its potential for high-temperature stealth applications. As the heating power exceeds 250 W, the Mo plate reaches a temperature above 424.4 °C and begins to gradually oxidize, resulting in higher emissivity and reduction of the temperature difference with the IR-selective emitter. If the reference maintained low emissivity (dashed line), more pronounced temperature reduction could be observed at higher input power, with a predicted temperature reduction of 114.9 °C at 600 W. Figure 4c shows the radiative temperatures of the IR-selective emitter and Mo plate at different input powers. Above 250 W, the radiative temperature of Mo plate rises sharply, and at around 600 W, the selective emitter exhibits a radiative temperature reduction of 103.4 °C lower than that of the Mo plate. Figure 4d displays LWIR images of the IR-selective emitter and Mo plate at input powers of 119 and 430 W, indicating that the IR-selective emitter exhibits better stealth performance at high temperatures, even though its radiation is slightly higher than that of the Mo plate at low temperatures.

### High-Temperature Microwave Stealth

**Fig. 5:**
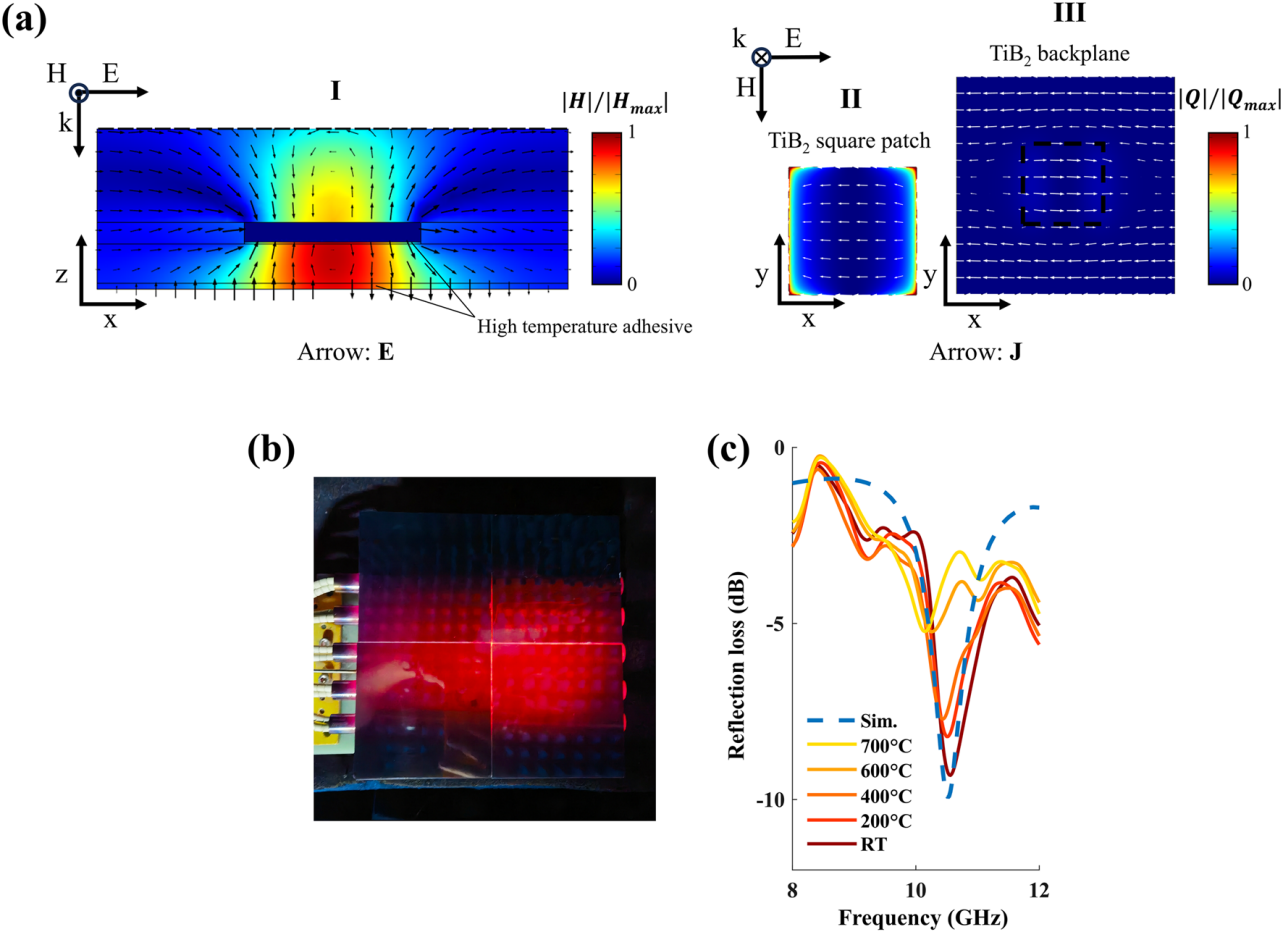
Simulations and demonstration of high-temperature microwave stealth. **a** Normalized magnetic field intensity (color) and electric field (arrow) on the cross section I (*x*–*z* plane, parallel to polarization); the normalized resistive loss (color) and surface currents (arrow) on the regions II and III (*x*–*y* plane, perpendicular to wave vector). The frequency is 10.5 GHz. **b** Image of the overall structure on the high-temperature ceramic heating stage at 700 °C. **c** The simulated reflection loss of the overall structure, as well as the measured reflection loss from room temperature (RT) to 700 °C. (Color figure online)

High-temperature microwave stealth is demonstrated through simulation and experiment. The simulation of the overall structure's microwave reflection loss in the X-band (8–12 GHz) is conducted under normally incident TM-polarized waves (Fig. 5). The TiB_2_-Al_2_O_3_-TiB_2_ microwave metasurface incorporates high-temperature adhesive during fabrication, which is also considered in the simulation. Region I in Fig. 5a shows the electromagnetic field distribution of minimum periodic unit at the resonant frequency ${f}_{0}$ = 10.5 GHz. The magnetic field is significantly enhanced in the Al_2_O_3_ interlayer of the microwave metasurface. Surface currents are induced on the TiB_2_ square blocks and TiB_2_ reflective backplate (white arrows in the regions II and III in Fig. 5a), leading to ohmic losses concentrated primarily on the boundaries of the TiB_2_ square blocks corresponding to a reflection loss peak of − 10 dB (Fig. 5b, blue dashed line).

The structure is heated from room temperature (RT) to 700 °C, and its reflection loss in the X-band is measured at different temperatures (Fig. 5c). As the temperature increases, it is observed that the absorption peak shifts toward lower frequencies and the absolute peak value decreases. This is primarily attributed to the temperature-dependent dielectric constant of Al_2_O_3_. According to the Debye equations [37], an increase in temperature leads to a reduction in the material's relaxation time, resulting in an increase in the real part of the dielectric constant of Al_2_O_3_, thereby altering the resonant frequency of the structure. At elevated temperatures, the change in the dielectric constant of Al_2_O_3_, combined with the reduced conductivity of TiB_2_ [47], degrades the impedance matching, resulting in decreased absorption. However, even at 700 °C, the structure maintains an effective absorption bandwidth of 9.6–12 GHz with absorption level lower than − 3 dB, demonstrating its potential for high-temperature microwave stealth.

## Conclusions

High-temperature multi-infrared and microwave stealth with enhanced thermal management is demonstrated through the integration of an IR-selective emitter and a microwave metasurface. First, our device achieves a maximum operating temperature and heat dissipation capabilities that surpass the current state of the art for simultaneous high-temperature IR and microwave stealth within the framework of photonic structures (Fig. S7). Second, it enables broad multispectral control by effectively manipulating five infrared bands and one microwave band, tailored to the spectral characteristics of high-temperature objects, thereby facilitating applications that require spectrum manipulation in extreme environments. Third, this device can be integrated with thermal insulation technologies to achieve higher operating temperatures and perform well under both internal and external heat sources. Last, the fabrication process of this device involves simple techniques such as deposition and laser etching, making it easy to implement and scalable for large-area applications. Ultimately, this work opens opportunities for thermal emission control [48–56], multispectral information processing [57, 58], and thermal management [59–61] in extreme environments, paving the way for robust and energy-efficient devices for both military and civilian applications.

## Supplementary Information

Below is the link to the electronic supplementary material.

Supplementary file 1
