# Supplementary material for: High-Temperature Stealth Across Multi-Infrared and Microwave Bands with Efficient Radiative Thermal Management
Source: Nanomicro Lett. 2025 Mar 24;17:199. doi: 10.1007/s40820-025-01712-5 (PMC11933609; doi:10.1007/s40820-025-01712-5)
Supplement: Supplementary file 1 [file 40820_2025_1712_MOESM1_ESM.docx]

Supporting Information for

**High-Temperature Stealth Across Multi-Infrared and Microwave Bands with Efficient Radiative Thermal Management**

Meng Zhao^1^, Huanzheng Zhu^1^, Bing Qin^1^, Rongxuan Zhu^1^, Jihao Zhang^1^, Pintu Ghosh^1^, Zuojia Wang^2^, Min Qiu^3^, and Qiang Li^1, *^

^1^ State Key Laboratory of Extreme Photonics and Instrumentation, College of Optical Science and Engineering, Zhejiang University, Hangzhou 310027, P. R. China

^2^ College of Information Science and Electronic Engineering, Zhejiang University, Hangzhou 310027, P. R. China

^3^ Key Laboratory of 3D Micro/Nano Fabrication and Characterization of Zhejiang Province, School of Engineering, Westlake University, Hangzhou 310024, P. R. China

[*Corresponding authors. E-mail: qiangli@zju.edu.cn](mailto:*Corresponding%20authors.%20E-mail:%20qiangli@zju.edu.cn) (Qiang Li)

**S1 Influence of laser etching process on stealth and thermal management**

The IR selective emitter composed of a multilayer film Al_2_O_3_ (17 nm)/Si (390 nm)/Mo (18 nm)/Si (700 nm)/Mo (50 nm)/Ti (10 nm) is deposited on two Si substrates, with one-piece laser-etched featuring line divisions that have a period of $l$= 500 μm and a line width of $w$= 15 μm. Laser etching achieves high precision, with a linewidth error of less than 1 μm (Fig. S1(b)). The emissivity after etching ($\epsilon_{a}$) can be estimated by:

$\epsilon_{a}=\epsilon_{b}f+\epsilon_{Si}(1-f)$ (S1)

$f={(\frac{l-w}{l})}^{2}$ (S2)

where $\epsilon_{b}$ is the emissivity before etching, $f$ is the filling ratio, and $\epsilon_{Si}$ is the emissivity of Si substrate. The laser-etched and unetched samples are placed onthe heating stage with temperature of 600 °C and observed using an MWIR infrared camera (Fig. S1(a)). The average signal intensity within the black dashed box for the etched sample is 6896, which showed almost no difference with that of the unetched sample, 6925. This can be attributed to the particularly fine line width of the etching, which has a negligible effect on emissivity. Therefore, the post-etching samples exhibited negligible changes in infrared stealth and radiative cooling performance compared to the pre-etching state.

**S2 Microwave metasurface design**

The microwave-absorbing metasurface structure adopted in this work is a typical metal-dielectric-metal configuration, and its electromagnetic response to microwaves can be analyzed using an equivalent circuit model. The metasurface structure can be equivalently represented as an *RLC* circuit comprising an inductor *L*, a capacitor *C*, and a resistor *R_L_+R_C_​*. The impedance *Z* can be expressed as:

$Z=j\omega L+\frac{1}{j\omega C}+R_{L}+R_{C}$(S3)

The resonant frequency is given by$:$

$f=\frac{1}{2\pi\sqrt{LC}}$ (S4)

and the reflection loss RL can be calculated as:

$RL=20log\left| \frac{Z-Z_{0}}{Z+Z_{0}} \right|$ (S5)

The precise expressions relating circuit parameters to metasurface parameters are relatively complex and can be calculated using the strip line analog [S1-S3]. In brief, the resonant frequency of the metasurface primarily depends on the thickness and permittivity of the dielectric layer, periodicity, and the side length of the TiB₂ square. By referencing the parameters used in existing studies on X-band microwave-absorbing metasurfaces[S4, S5] and performing parameter sweep through COMSOL Multiphysics simulations, we obtained metasurface parameters that exhibit a resonant frequency within the X-band, high absorption, and a relatively broad bandwidth.

We conducted parameter sweeps as follows: the side length *a* of the TiB₂ square was varied from 3 to 7 mm, the periodicity *p* of the metasurface was scanned from 9 to 15 mm, and the thickness *h_3_* of the intermediate Al₂O₃ layer was varied from 0.5 to 1.5 mm. The initial parameters were set to *a* = 5 mm, *p* = 12 mm, and *h_3_* = 1 mm. The relationship between the absorptivity of the microwave metasurface in 8–12 GHz and these parameters is shown in Fig. S2 (a-c). It is evident that the resonant frequency of the metasurface is primarily determined by the side length *a* of the TiB₂ square, while the thickness *h_3_* of the intermediate Al₂O₃ layer and the periodicity *p* have a minor influence on the resonant frequency. The maximum absorptivity exhibits low sensitivity to these three parameters, consistently remaining above 0.8. To ensure that the absorption covers the X-band as much as possible while maintaining a high peak absorptivity, we selected *a* = 4.5 mm, *p* = 12 mm, and *h_3_* = 1 mm, which places the absorption peak near 10 GHz with a maximum absorptivity close to 1.

Next, we investigated the influence of the thickness *h_1_* of the IR-selective emitter substrate on microwave absorption. By sweeping *h_1_* from 1.5 to 2.4 mm, we found that the microwave absorptivity remains above 0.8 (Fig. S2(d)). However, as the thickness increases, the absorption peak becomes higher but narrower. To balance high absorptivity and relatively broad bandwidth, we chose *h_1_* = 2.3 mm.

**S3 Supplementary Tables and Figures**

The average emissivity for the MWIR and LWIR bands, as well as the non-atmospheric window of 5-8 μm (Table S1) is calculated using the following equation:

$\bar{\epsilon}=\frac{\int_{\lambda_{1}}^{\lambda_{2}} \epsilon(\lambda)u(\lambda,T)}{\int_{\lambda_{1}}^{\lambda_{2}} u(\lambda,T)}$ (S9)

where $\epsilon(\lambda)$ is the emissivity spectrum, $\lambda_{1}$ and $\lambda_{2}$ represent the band ranges, $u(\lambda,T)$ represent the blackbody radiation energy density.

**Table S1** The average emissivity of the IR selective emiter in MWIR and LWIR bands, and 5-8 μm band for temperatures from 200 to 800 °C

| Temperature (°C) | | ${\bar{\boldsymbol{\epsilon}}}_{\mathbf{3-5}\boldsymbol{\mu m}}$ | ${\bar{\boldsymbol{\epsilon}}}_{\mathbf{5-8}\boldsymbol{\mu m}}$ | ${\bar{\boldsymbol{\epsilon}}}_{\mathbf{8-14}\boldsymbol{\mu m}}$ |
| --- | --- | --- | --- | --- |
| 200 | 0.40 | | 0.74 | 0.292 |
| 300 | 0.36 | | 0.76 | 0.33 |
| 400 | 0.33 | | 0.77 | 0.36 |
| 500 | 0.36 | | 0.79 | 0.40 |
| 600 | 0.37 | | 0.80 | 0.42 |
| 650 | 0.36 | | 0.80 | 0.43 |
| 700 | 0.38 | | 0.82 | 0.44 |
| 750 | 0.61 | | 0.71 | 0.68 |
| 800 | 0.60 | | 0.69 | 0.67 |


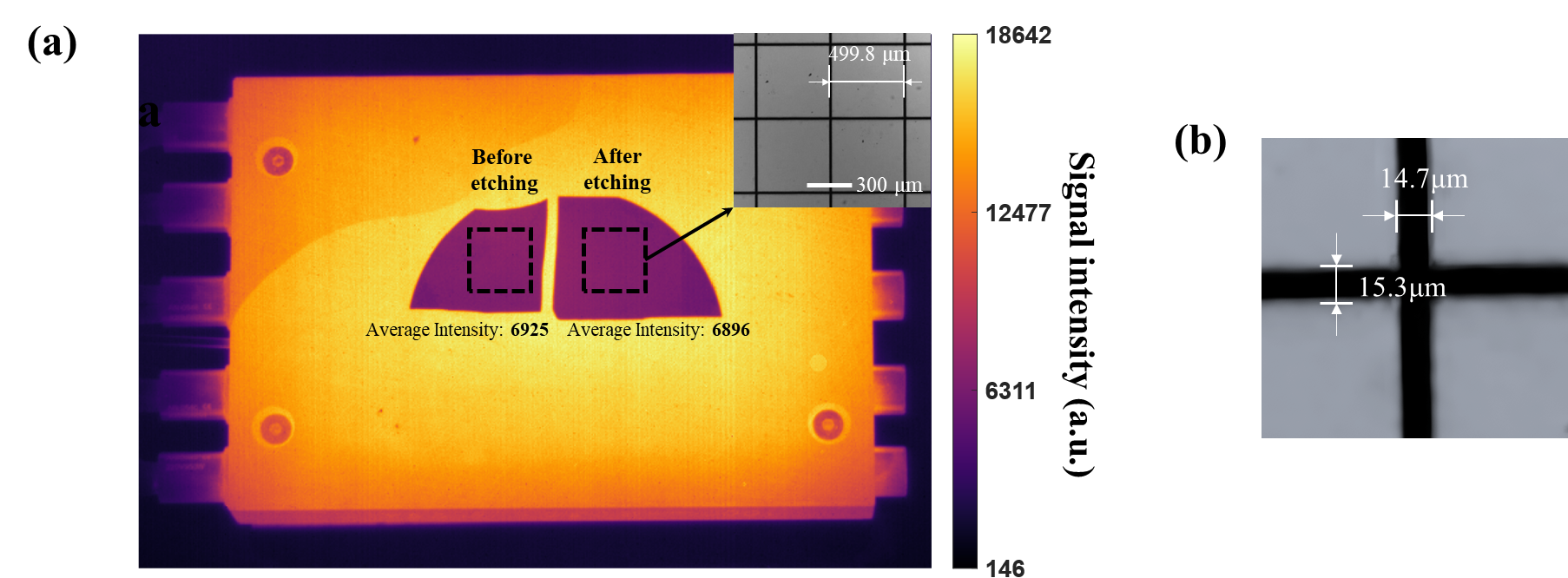


**Fig. S1** (**a**) MWIR images of laser-etched sample (microscope image in upper right corner) and unetched sample at a heating temperature of 600°C. (**b**) Linewidth of laser-etched sample


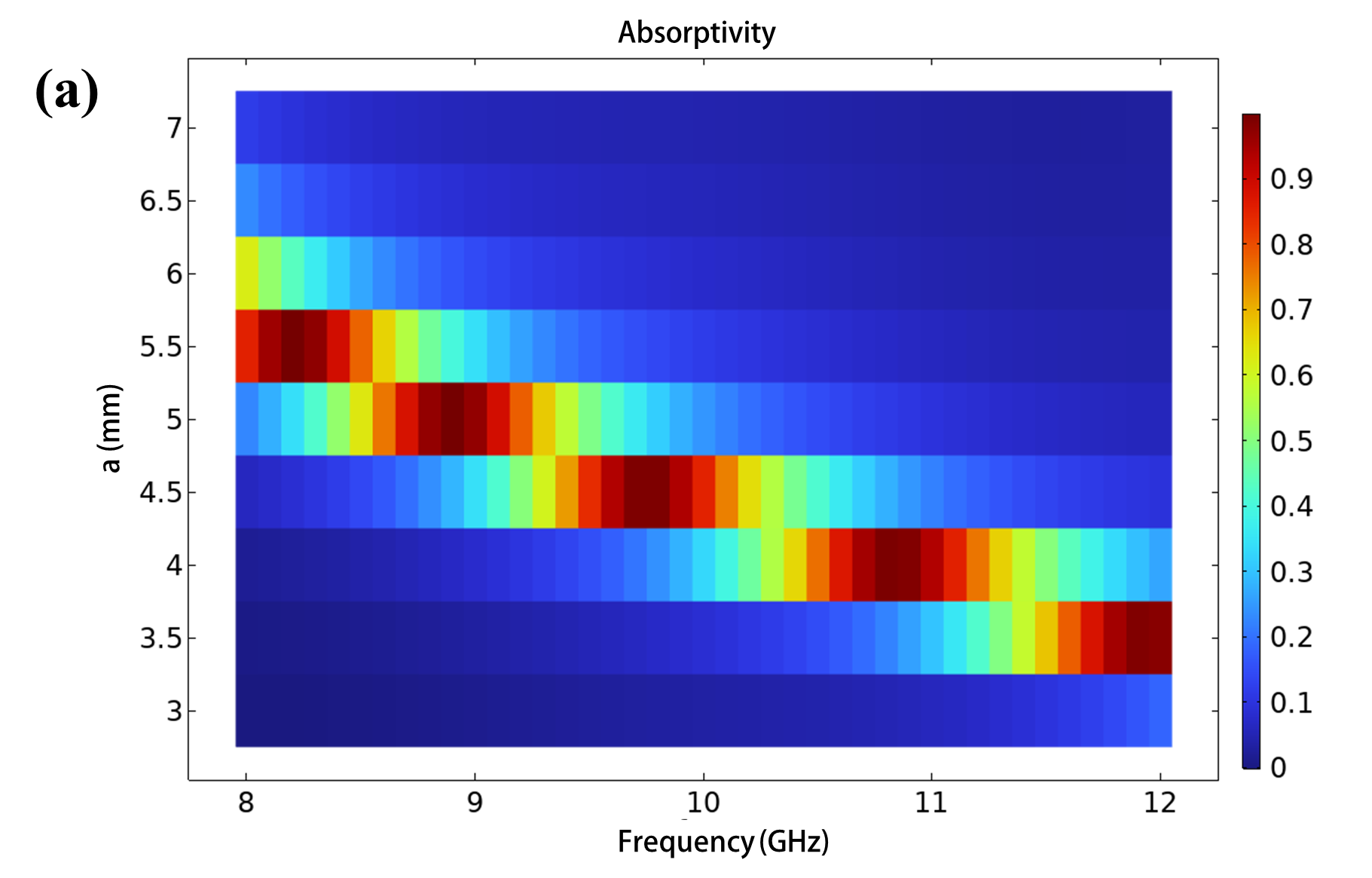

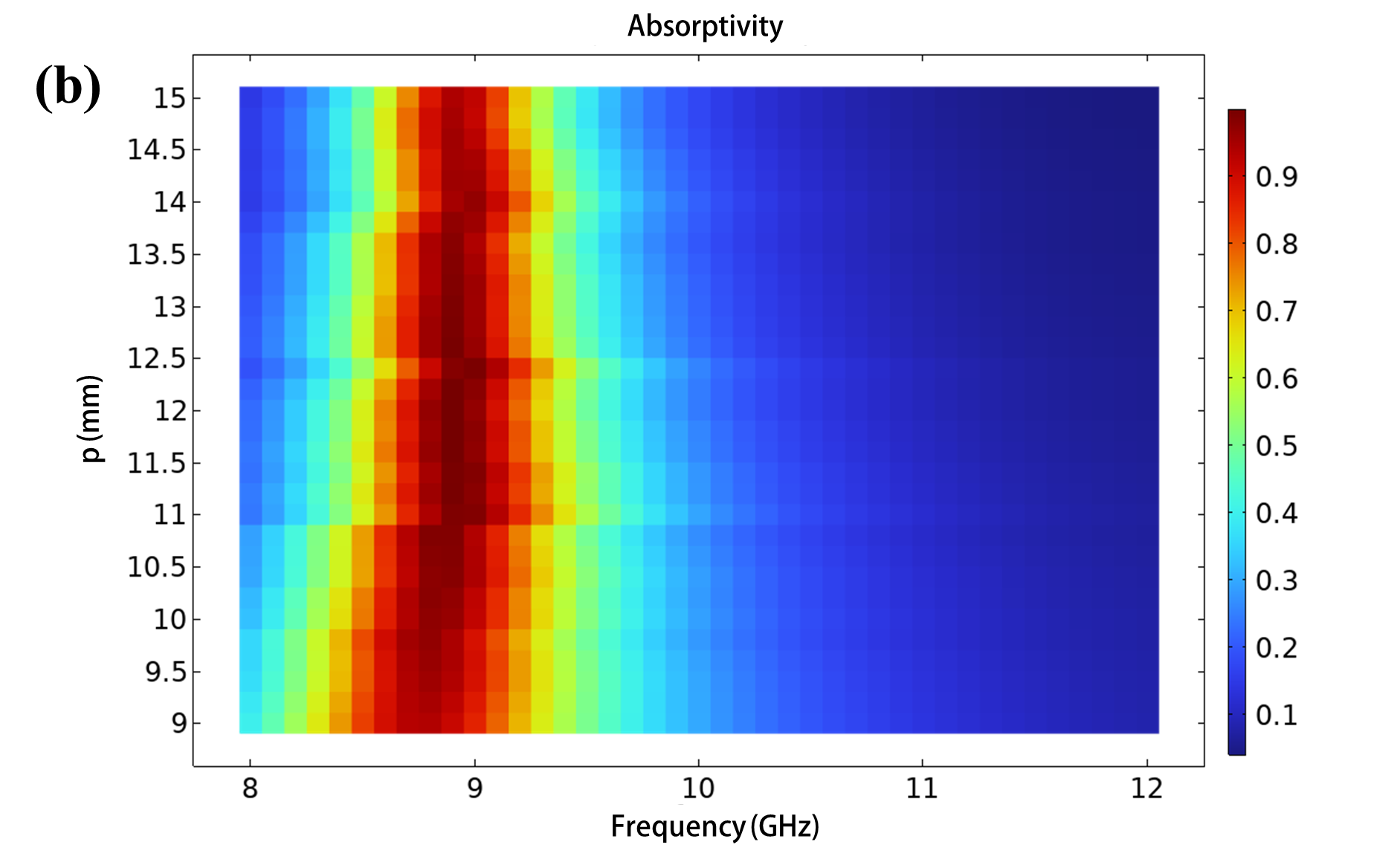


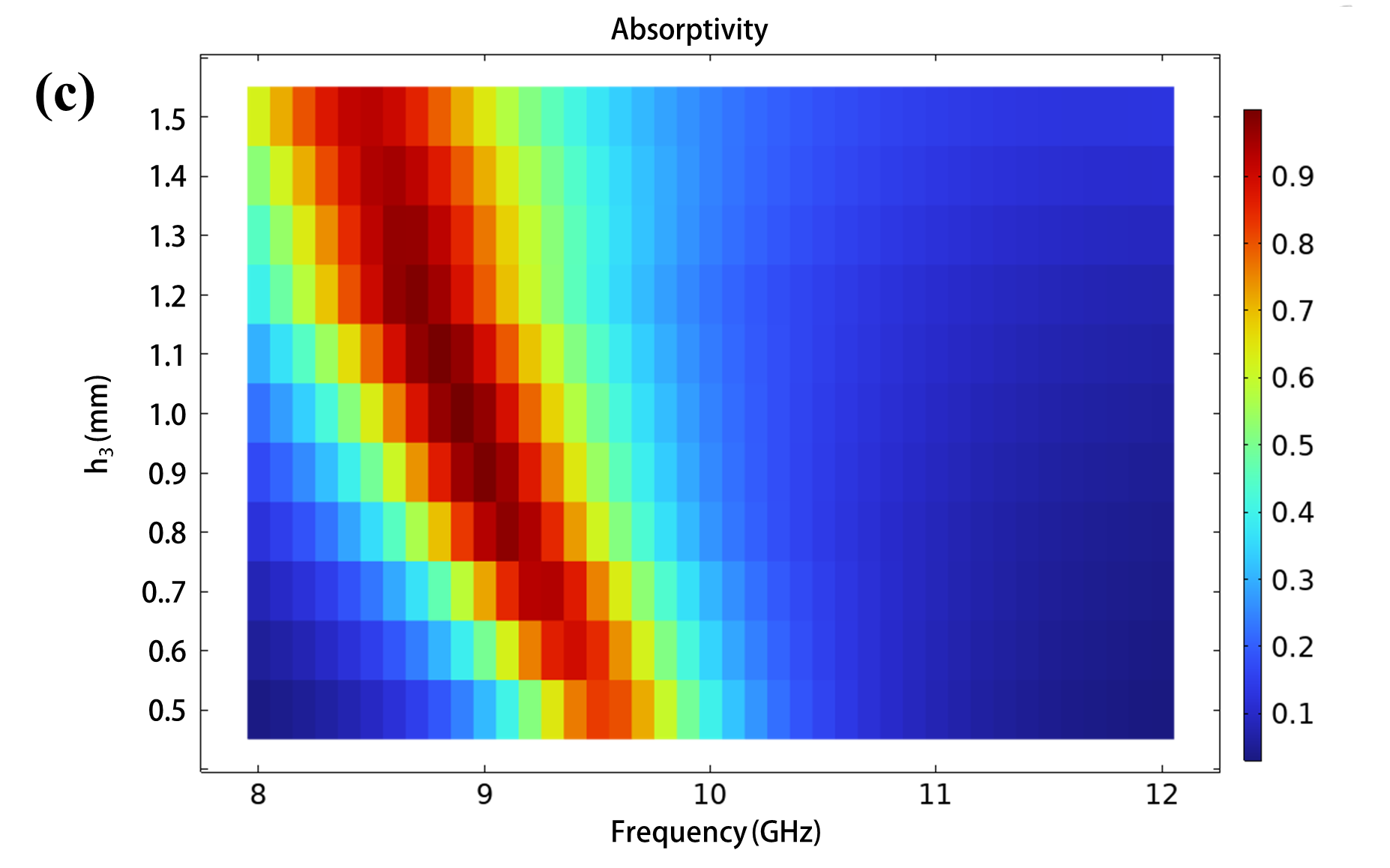

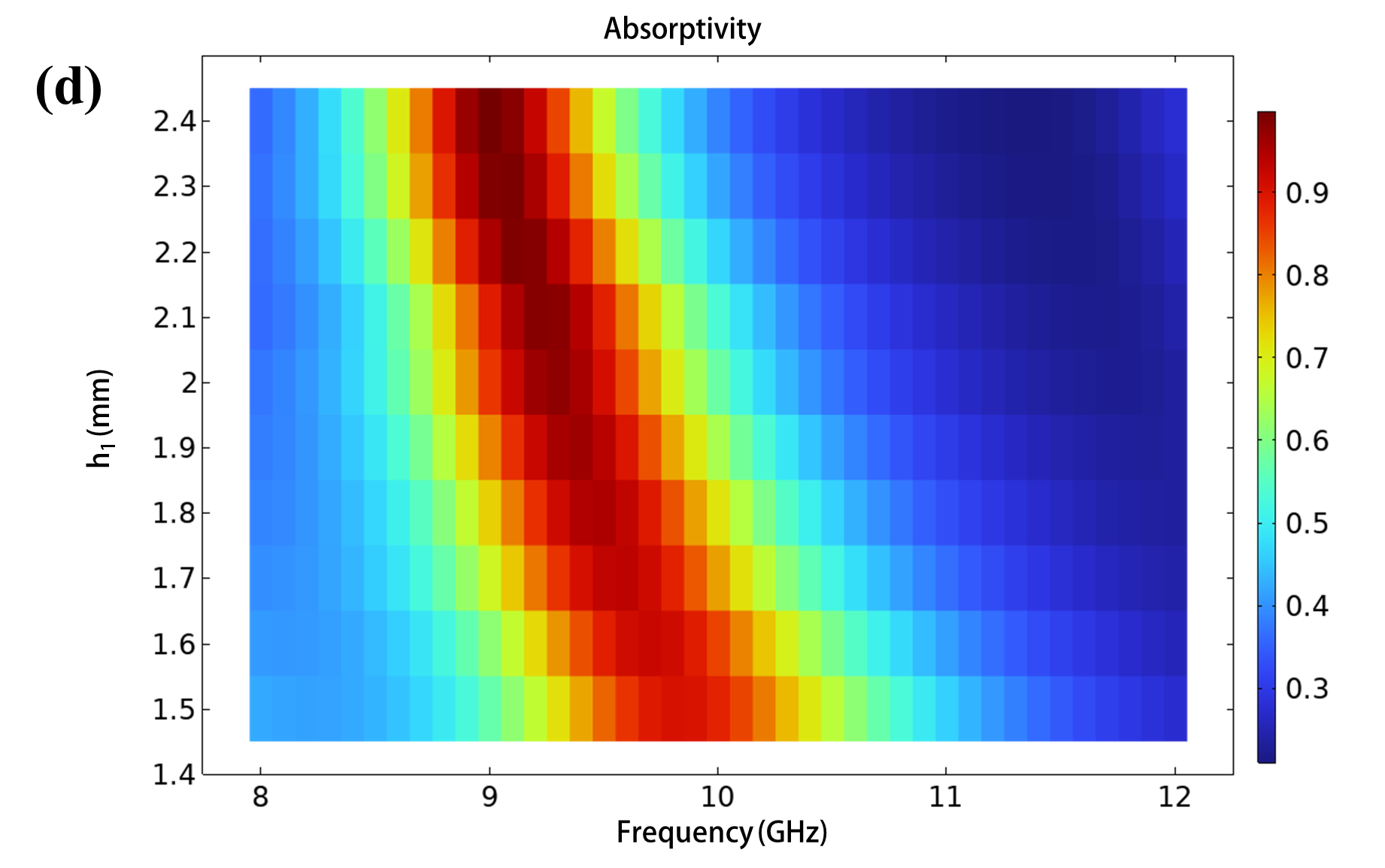


**Fig. S2** (**a**) The influence of the side length a of TiB₂ squares, (**b**) the periodicity p, and (**c**) the thickness h3 of the intermediate Al₂O₃ layer on the absorptivity of the microwave metasurface. (**d**) The effect of the substrate thickness h1 of the IR-selective emitter on the absorptivity


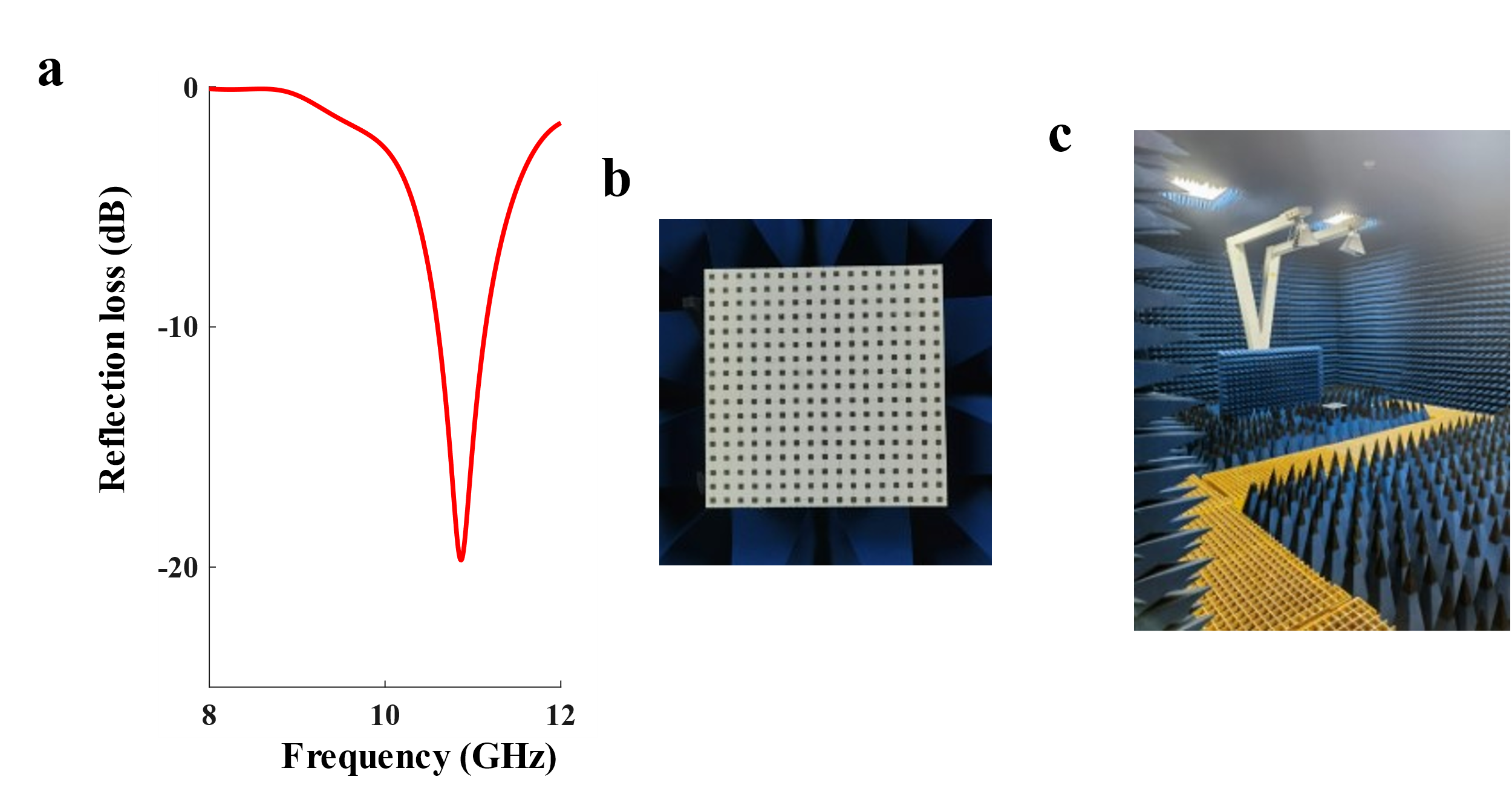


**Fig. S3** (**a**) Measured reflection loss of the microwave metasurface. (**b**) Photograph of the microwave metasurface. (**c**) Arch-shaped reflectivity measurement device


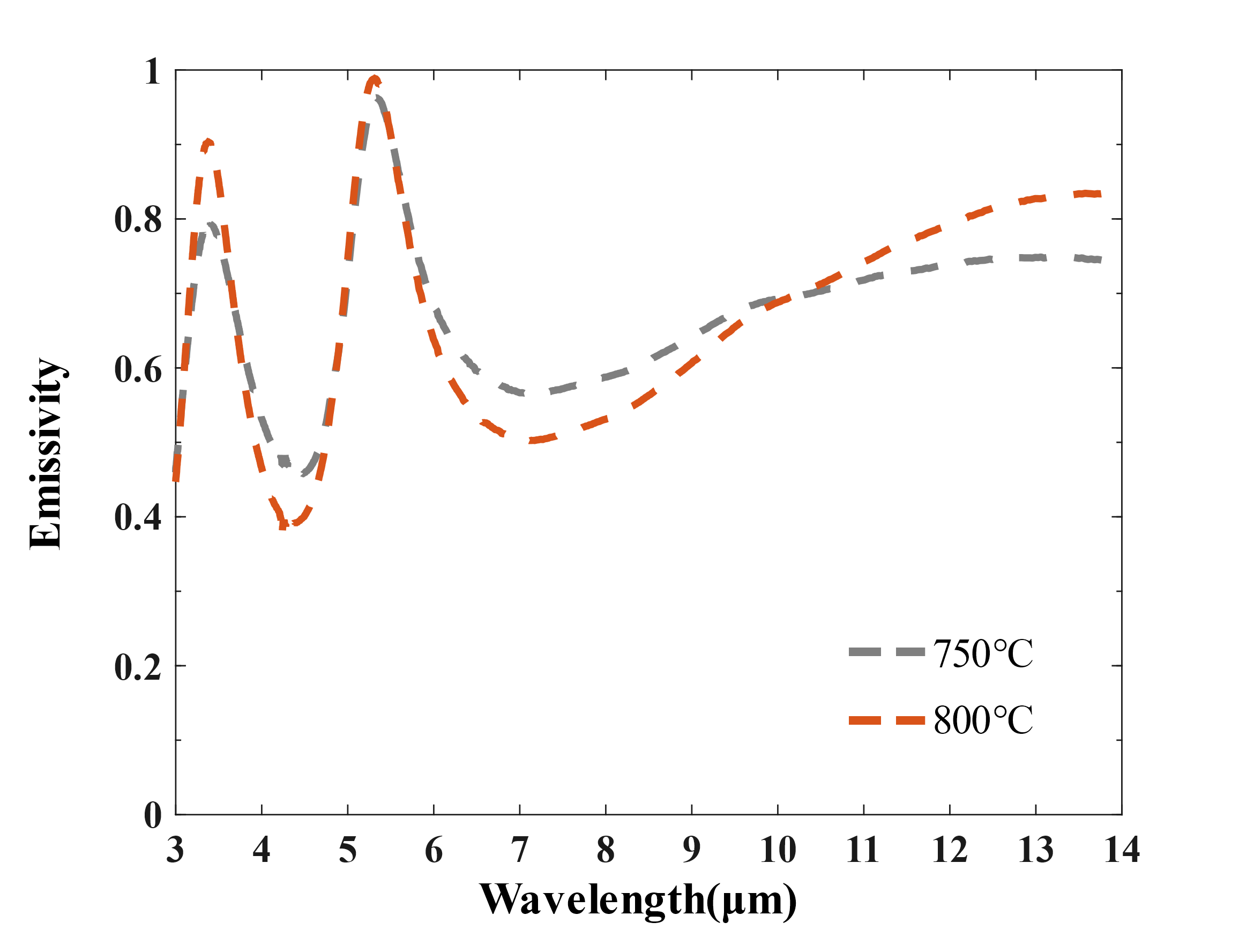


**Fig. S4** Measured emissivity of the IR selective emitter at 750 °C and 800 °C
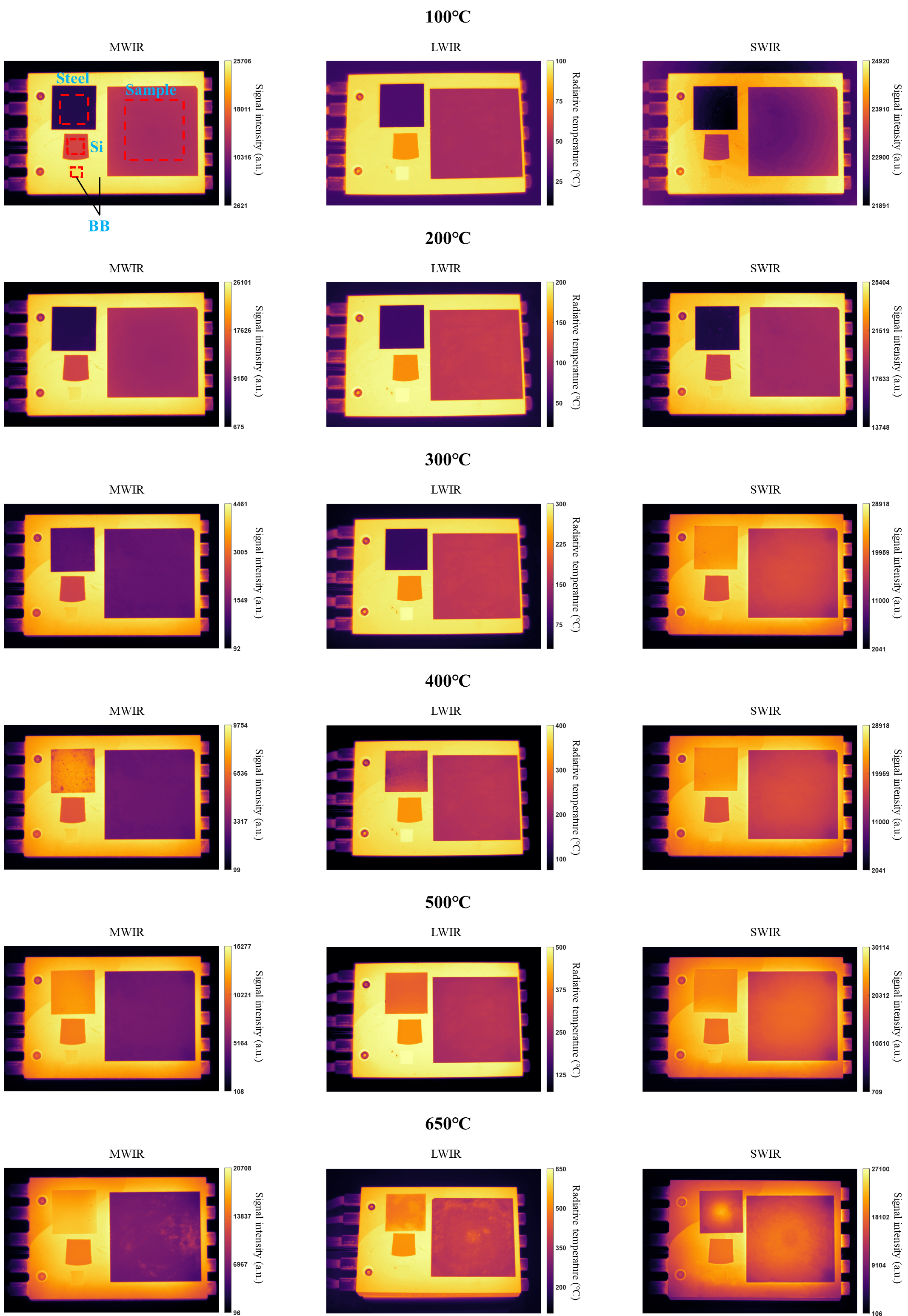


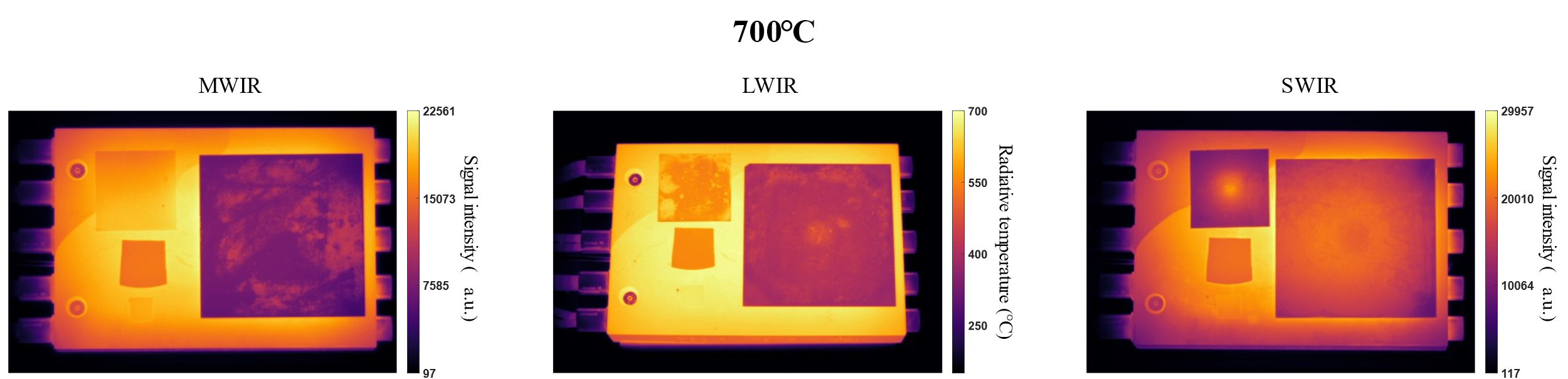


**Fig. S5** Thermal image of the IR selective emitter, a steel plate, a silicon wafer, a BB in the MWIR, LWIR, and SWIR bands at 100, 200, 300, 400, 500, 650, and 700 °C


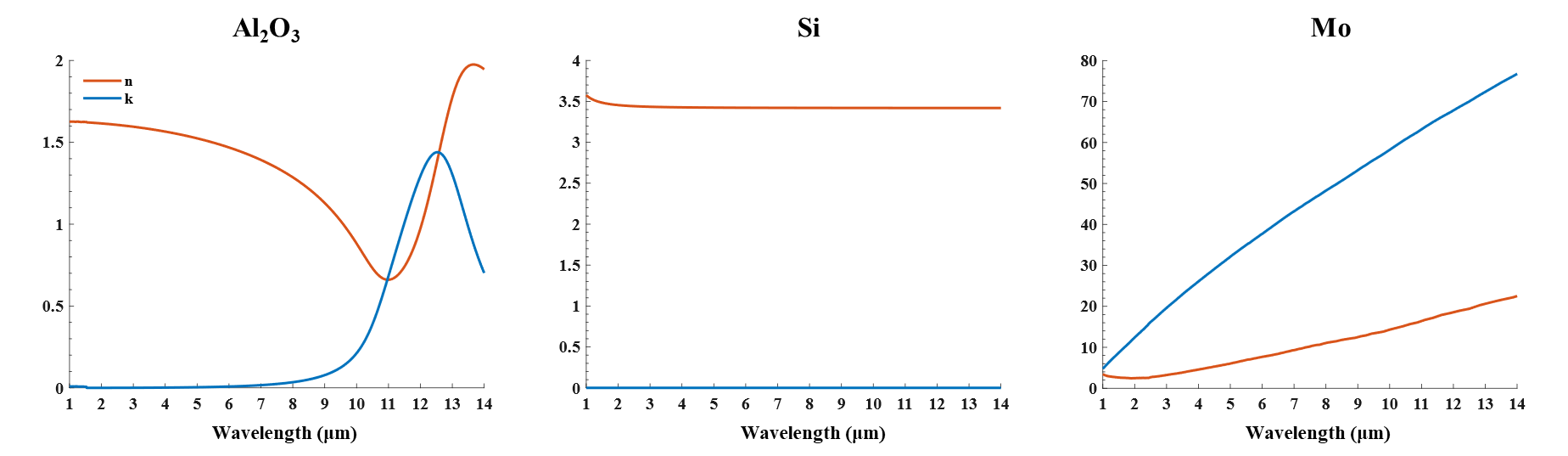


**Fig. S6** Refractive index (n) and extinction coefficient (k) of the Al_2_O_3_ [S6], Si [S7], and Mo [S8]


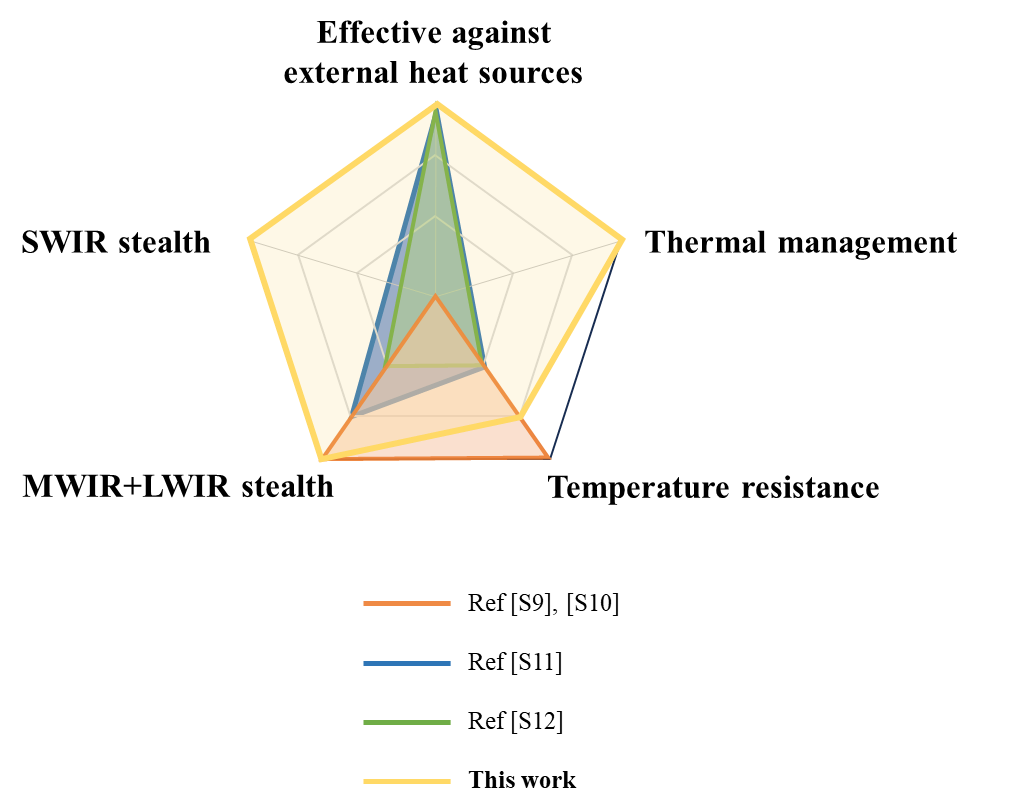


**Fig. S7** Comparison with previous works on high-temperature simultaneous IR and microwave stealth

**Supplementary References**

1. F. Costa, S. Genovesi, A. Monorchio, G. Manara, A circuit-based model for the interpretation of perfect metamaterial absorbers. IEEE Trans. Antennas Propag. **61**, 1201–1209 (2013). <https://doi.org/10.1109/TAP.2012.2227923>
2. P.K. Singhal, S. Mathur, R.N. Baral, Compact narrow band non-degenerate dual-mode microstrip filter with etched square lattices. J. Electromagn. Anal. Appl. **2**, 98–103 (2010). <https://doi.org/10.4236/jemaa.2010.22014>
3. F. Costa, A. Monorchio, G. Manara, Efficient analysis of frequency-selective surfaces by a simple equivalent-circuit model. IEEE Antennas Propag. Mag. **54**, 35–48 (2012). <https://doi.org/10.1109/MAP.2012.6309153>
4. Y. Zhu, L. Zhang, J. Wang, B. Feng, M. Feng et al., Microwave-infrared compatible stealth via high-temperature frequency selective surface upon al2o3-tic coating. J. Alloy. Compd. **920**, 165977 (2022). <https://doi.org/10.1016/j.jallcom.2022.165977>
5. Y. Li, W. Li, Y. Wang, J. Cao, J. Guan, Refractory metamaterial microwave absorber with strong absorption insensitive to temperature. Adv. Opt. Mater. **6**, 1800691 (2018). <https://doi.org/10.1002/adom.201800691>
6. M.R. Querry, Optical constants. Contractor report (1985).
7. D. Franta, A. Dubroka, C. Wang, A. Giglia, J. Vohánka et al., Temperature-dependent dispersion model of float zone crystalline silicon. Appl. Surf. Sci. **421**, 405–419 (2017). <https://doi.org/10.1016/j.apsusc.2017.02.021>
8. M.M. KffiiLLOVA, L.V. Nomerovannaya, M.M. Noskov, Optical properties of molybdenum single crystals. Sov. Phys. JETP 33, (1971).
9. Z. An, Y. Huang, R. Zhang, High-temperature multispectral stealth metastructure from the microwave-infrared compatible design. Compos. Part B Eng. **259**, 110737 (2023). <https://doi.org/10.1016/j.compositesb.2023.110737>
10. Z. An, Y. Li, X. Luo, Y. Huang, R. Zhang et al., Multilaminate metastructure for high-temperature radar-infrared bi-stealth: topological optimization and near-room-temperature synthesis. Matter **5**, 1937–1952 (2022). <https://doi.org/10.1016/j.matt.2022.04.011>
11. Y. Zhu, L. Zhang, J. Wang, F. Zhou, B. Gui et al., High-entropy alloy: an alternative approach for high temperature microwave-infrared compatible stealth. Ceram. Int. **49**, 25576–25584 (2023). <https://doi.org/10.1016/j.ceramint.2023.05.098>
12. B. Gui, J. Wang, Y. Zhu, L. Zhang, M. Feng et al., High temperature infrared-radar compatible stealthy metamaterial based on an ultrathin high-entropy alloy. Opt. Express **30**, 45426 (2022). <https://doi.org/10.1364/OE.475355>
